# Supplementary material for: High-density electrode recordings reveal strong and specific connections between retinal ganglion cells and midbrain neurons
Source: Nat Commun. 2022 Sep 5;13:5218. doi: 10.1038/s41467-022-32775-2 (PMC9445019; doi:10.1038/s41467-022-32775-2)
Supplement: Supplementary file 1 — Supplementary Information [file 41467_2022_32775_MOESM1_ESM.pdf]

High-density electrode recordings reveal strong and specific connections between retinal ganglion cells and midbrain neurons (Supplementary Figures)

Jérémie Sibille[1,2,3,4], Carolin Gehr[1,2,3,4], Jonathan I. Benichov[5,6], Hymavathy Balasubramanian[1,2,3,4], Kai Lun Teh[1,2,3,4], Tatiana Lupashina[1,2,3,4], Daniela Vallentin[5,6], Jens Kremkow[1,2,3,4*]

[1]Neuroscience Research Center, Charité-Universitätsmedizin Berlin, Charitéplatz 1,10117 Berlin, Germany
[2]Bernstein Center for Computational Neuroscience Berlin, Philippstraße 13, 10115 Berlin, Germany
[3]Institute for Theoretical Biology, Humboldt-Universität zu Berlin, Philippstraße 13, 10115 Berlin, Germany
[4]Einstein Center for Neurosciences Berlin, Charitéplatz 1,10117 Berlin, Germany
[5]Max Planck Institute for Ornithology, Eberhard-Gwinner Straße, 82319 Seewiesen, Germany
[6]Max Planck Institute for Biological Intelligence (in foundation), Eberhard-Gwinner Straße, 82319 Seewiesen, Germany

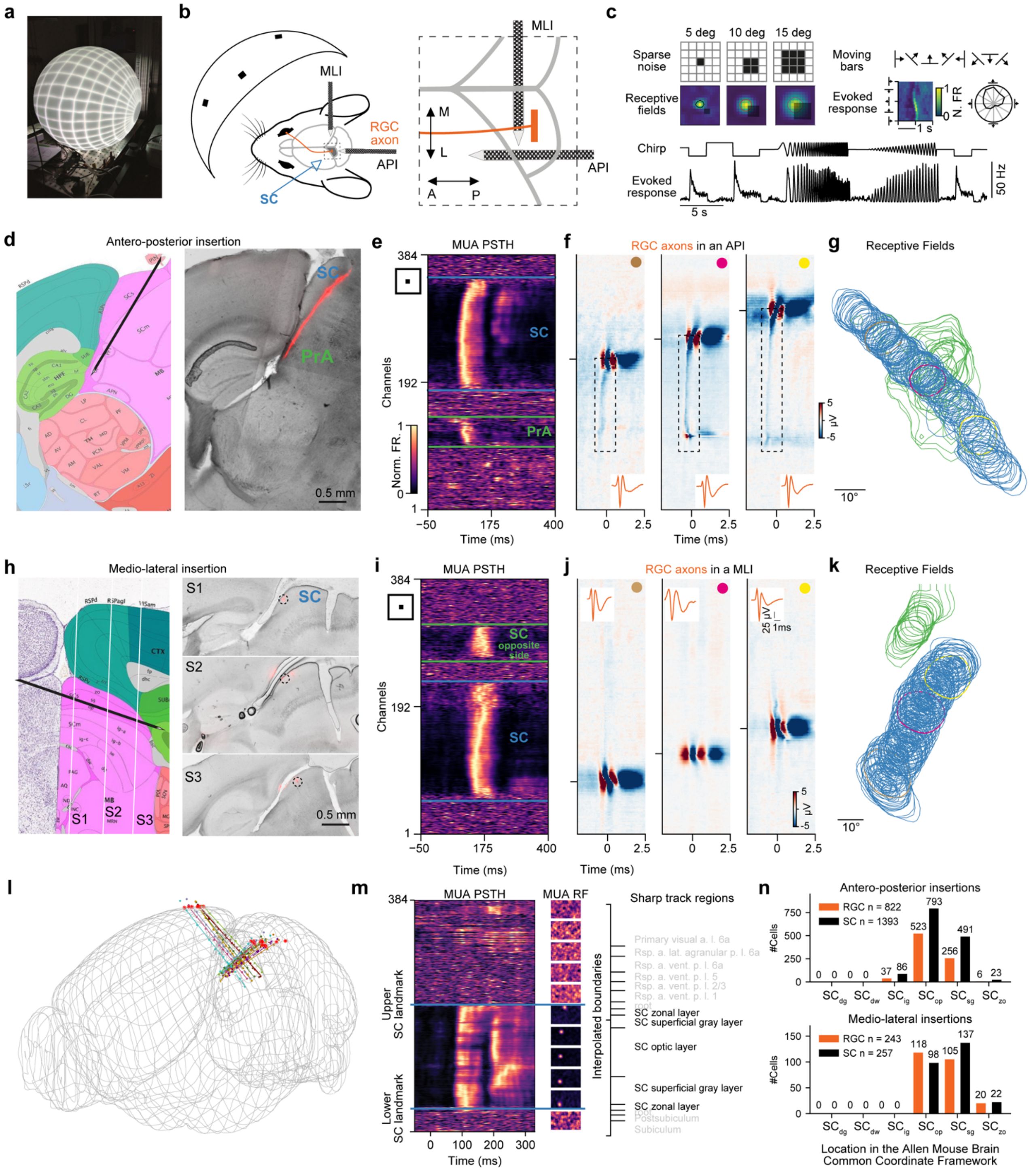

Fig. S1. Recording RGC axons and SC neurons in the mouse *in vivo* with high-density electrodes.
Legend on the next page.

Fig. S1. Recording RGC axons and SC neurons in the mouse *in vivo* with high-density electrodes. a Picture of the visual dome used in this study, adapted from[1]. **b** Schematic of the experimental setup. The mouse in the visual dome setup together with the antero-posterior and medio-lateral recording configurations and the axonal projections of retinal ganglion cells (RGC) to the SC is shown (left). Zoom onto the Neuropixels probe implantations along the antero-posterior axis (API) and medio-lateral axis (MLI) (right). **c** Visual stimuli used in this study to characterize the functional response properties. Receptive fields were mapped with sparse noise using targets of 5, 10 or 15 deg in size and presented on a 5 deg grid. Moving bars were used to measure orientation and direction tuning. A full-field chirp stimulus[2] was used to characterize the contrast and temporal response properties. **d** Sagittal brain slice with a DiI staining of the Neuropixels recording track from an antero-posterior insertion (n = 17 mice). The corresponding location in the Allen Mouse Brain Common Coordinate Framework (CCF) is shown on the left. **e** Visually evoked multi-unit-activity (MUA) to white sparse noise on black background from the recording in c. The peri-stimulus time histograms (PSTH) of all 384 channels are shown as a colormap. The SC is located in the upper part of the Neuropixels probe, between the blue horizontal lines. The visually evoked activity in the lower part of the probe originates from pretectal areas (PrA) (green). **f** Multi-channel waveforms (MCW) of three individual RGC axons from the recording shown in e. The dashed black rectangle highlights the action potential propagating along the axonal path. The best channel, i.e. the channel with the largest waveform amplitude, is indicated by the horizontal tick, and the insets show the RGC axonal waveforms at their respective best channels in orange. **g** Receptive fields of the multi-unit-activity in each visually driven channel: SC (blue) and PrA (green). Receptive fields at the best channel of the example single units shown in f are plotted in their respective colors. **h-k** Example recording along the medio-lateral axis (n = 7 mice), same format as for d-g. **h** Three consecutive sagittal slices (S1, S2, S3) with their coordinates marked by white lines in the Allen Mouse Brain Atlas are shown (left). The electrode track is highlighted by dashed circles in the histology images. In the medio-lateral recording configuration, the probe can pass through the SC on the opposite site. Note, the RGC action potential propagation is not visible in the medio-lateral recording configuration because the Neuropixels probe is not aligned with the RGC axons paths. **l** 3D illustration of the recording locations within the Allen Mouse Brain Common Coordinate Framework. Neuropixels electrode tracks were reconstructed and analyzed with SHARP-track[3,4]. **m** Example of SHARP-track analysis. Visually driven multi-unit-activity (left) together with the estimated SHARP-track locations (right). The entry and the exit of the Neuropixels probe in SC were used as physiological landmarks in the alignment step. **n** Histograms of the single unit SHARP-track locations in the antero-posterior (top) and medio-lateral insertions (bottom). Note that the majority of RGC axons are located in the SC optical layer and SC superficial gray layer.

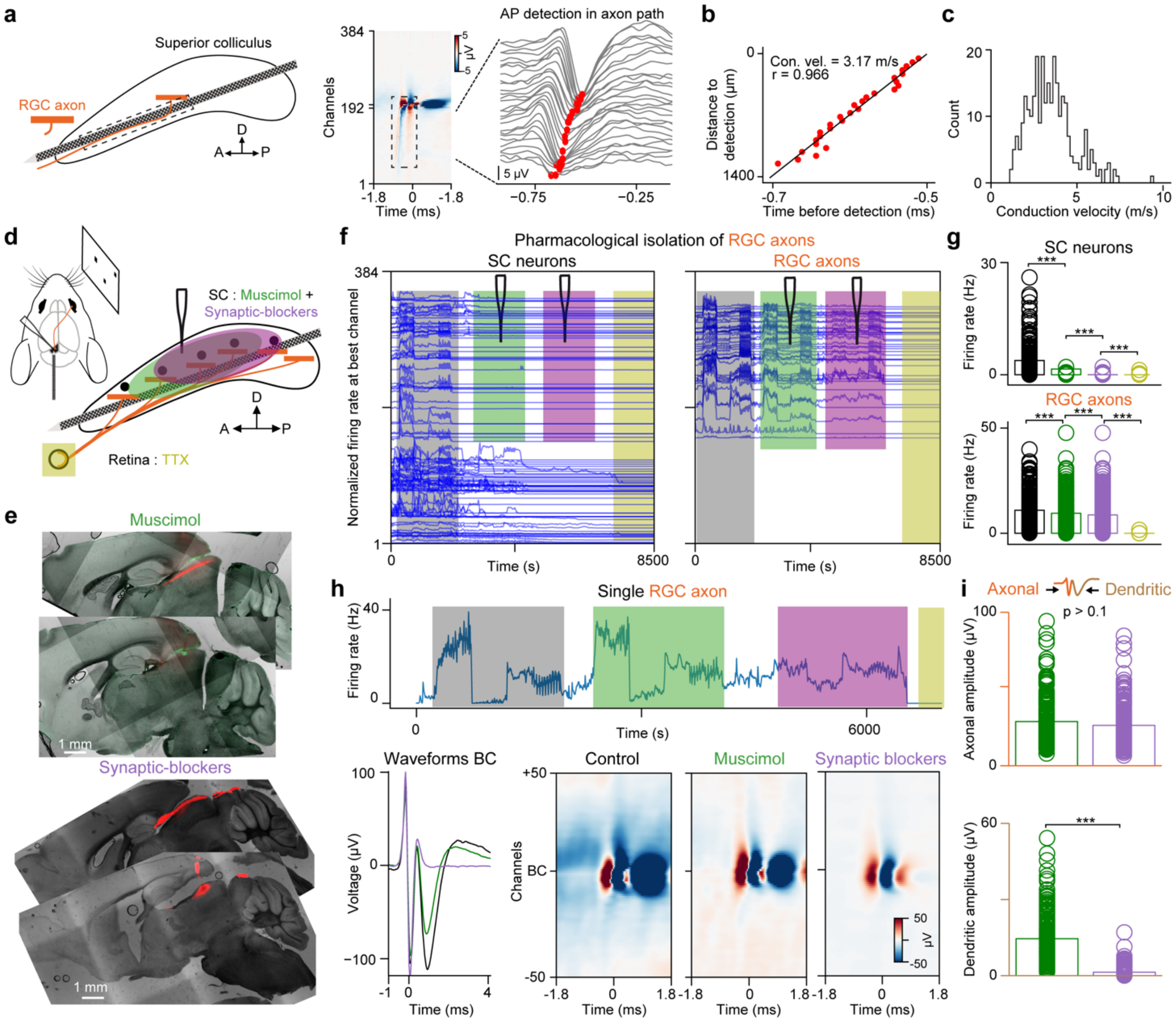

Fig. S2. Evidence that tri-phasic waveforms are RGC axons.
Legend on the next page.

Fig. S2. Evidence that tri-phasic waveforms are RGC axons. a RGC axonal action potential conduction velocity estimation. In the antero-posterior insertions the action potential propagation along the axon path (left, dashed rectangle) can be detected in the multi-channel waveforms (middle). Close up view on the propagating action potential together with the detection of the action potential by the local minimum of the waveform in each channel (right, red dots). **b** The conduction velocity was estimated from a linear fit to the action potential timepoints along the axon path (red dots in a, right). **c** Histogram of RGC axon conduction velocities across multiple recordings (mean conduction velocity = 3.5±1.3 m/s, n = 283 RGC axons, for RGC axons with R^2 of linear fit > 0.8). **d** Schematic of the pharmacological injections: muscimol (green), synaptic blocker (purple) and Tetrodotoxin (TTX, yellow). **e** Sagittal brain slices illustrating the injections of muscimol (top, green) and synaptic blocker (bottom, red) with DiI staining from antero-posterior insertions (red, n = 3 mice). **f** Activity of SC neurons and RGC axons throughout the entire recording duration aligned to their best channel. The pharmacological injections are indicated by different colors. Visually evoked activity of SC neurons decreases following muscimol injection, while the evoked activity of RGC axons remains unaffected. **g** Quantification of firing rate changes of SC neurons and RGC axons during the different pharmacological conditions. Control (black): SC neurons = 3.8±4.3 Hz, RGC axons = 10.6±7.9 Hz; muscimol (green): SC neurons = 1.5±2.6 Hz, RGC axons = 9.3±7.2 Hz; synaptic blocker (purple): SC neurons = 0.03±0.18 Hz, RGC axons = 8.5±8.6 Hz, TTX (yellow): SC neurons = 0.01±0.13 Hz, RGC axons = 0.008±0.1 Hz. *** $p = 5.7\times10^{-26}/4.33\times10^{-36}/2.47\times10^{-11}$, Cohen's d = 0.63/0.79/0.16, in n = 224 SC neurons. $p = 0.0001/5.40\times10^{-05}/4.99\times10^{-37}$, Cohen's d = 0.18/0.25/1.88 in n = 215 RGC axons. Two-sided Wilcoxon signed-rank test. **h** A single RGC axon during the entire recording (top). The waveform of the same RGC axons is shown at its best channel (bottom left) and across 100 channels (bottom right) for the control (black), muscimol (green) and synaptic blockade (purple) condition. Note that the postsynaptic dendritic rebound (second trough in the RGC axon waveform) is abolished after the application of synaptic blockers. **i** Quantification of the axonal (top) and dendritic (bottom) signal amplitudes during the application of muscimol (green) and synaptic blockers (purple) (axonal amplitude: muscimol = 28.8±14.5 μV, synaptic blocker = 26.3±13 μV, p > 0.1; dendritic amplitude: muscimol = 14.5±9.04 μV, synaptic blocker = 1.39±1.9 μV; n = 203 RGC axons). *** $p = 2.07\times10^{-51}$ using two-sided Student paired t-test. Source data are provided as a Source Data file.

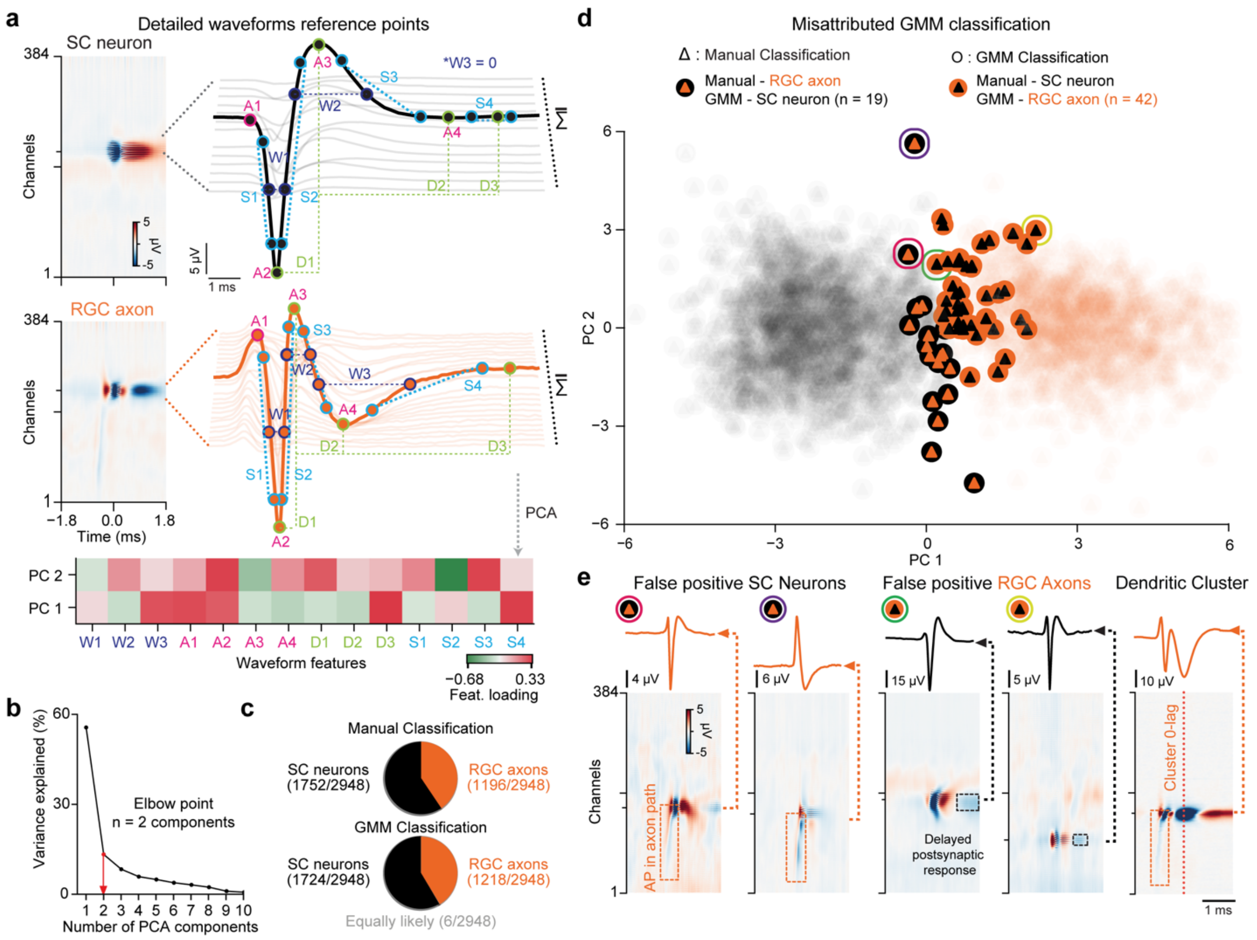

Fig. S3. RGC axon and SC neuron classification based on their waveforms. a Example waveforms from a SC neuron (top) and from an RGC axon in SC (middle). The multi-channel waveforms are shown as colormaps together with traces of several single-channel waveforms within the spatial spread of interest. The waveforms were characterized by the following measurements: W1 : half peak width of the detected negative peak (DNP), W2 : half peak width of positive peak after detection (PPAD), W3 : half peak width of second negative peak after detection (SNPAD), A1 : amplitude of the positive peak before detection (PPBD), A2 : DNP's amplitude, A3 : PPAD's amplitude, A4 : SNPAD's amplitude, D1 : difference DNP to PPAD, D2 : difference DNP to SNPAD, D3 : difference DNP to baseline, S1 : depolarization slope, S2 : repolarization slope, S3 : recovery slope PPAD to SNPAD, S4 : recovery slope SNPAD to baseline. The loading of these different features on the two first principal components (PCs) is represented in a heatmap (bottom). The two first PCs were used in the Gaussian mixture model (GMM) for classification. **b** PCA scree plot. The elbow point identifies n = 2 as the optimal number of components that was used as input to the Gaussian Mixture Model (GMM). **c** Pie charts showing the results of the GMM vs. the manual classification. **d** Scatter plot of PC1 and PC2 projections (top) of the manual classification (blurred). Misclassified GMM clusters are shown as large colored disks. Four examples (purple, red, green, yellow circle on top, corresponding to the examples in e) illustrate misclassified clusters. **e** False positive SC neurons are identified by the presence of an axonal streak (orange dashed squares) in the multi-channel-waveforms (two left). False positive RGC axons are identified by the presence of an unexpected negative peak after detection (black dashed squares two examples in the middle right). Waveforms that were detected on the postsynaptic response of the RGC axon waveform were identified as dendritic clusters and excluded from the dataset (right). Source data are provided as a Source Data file.

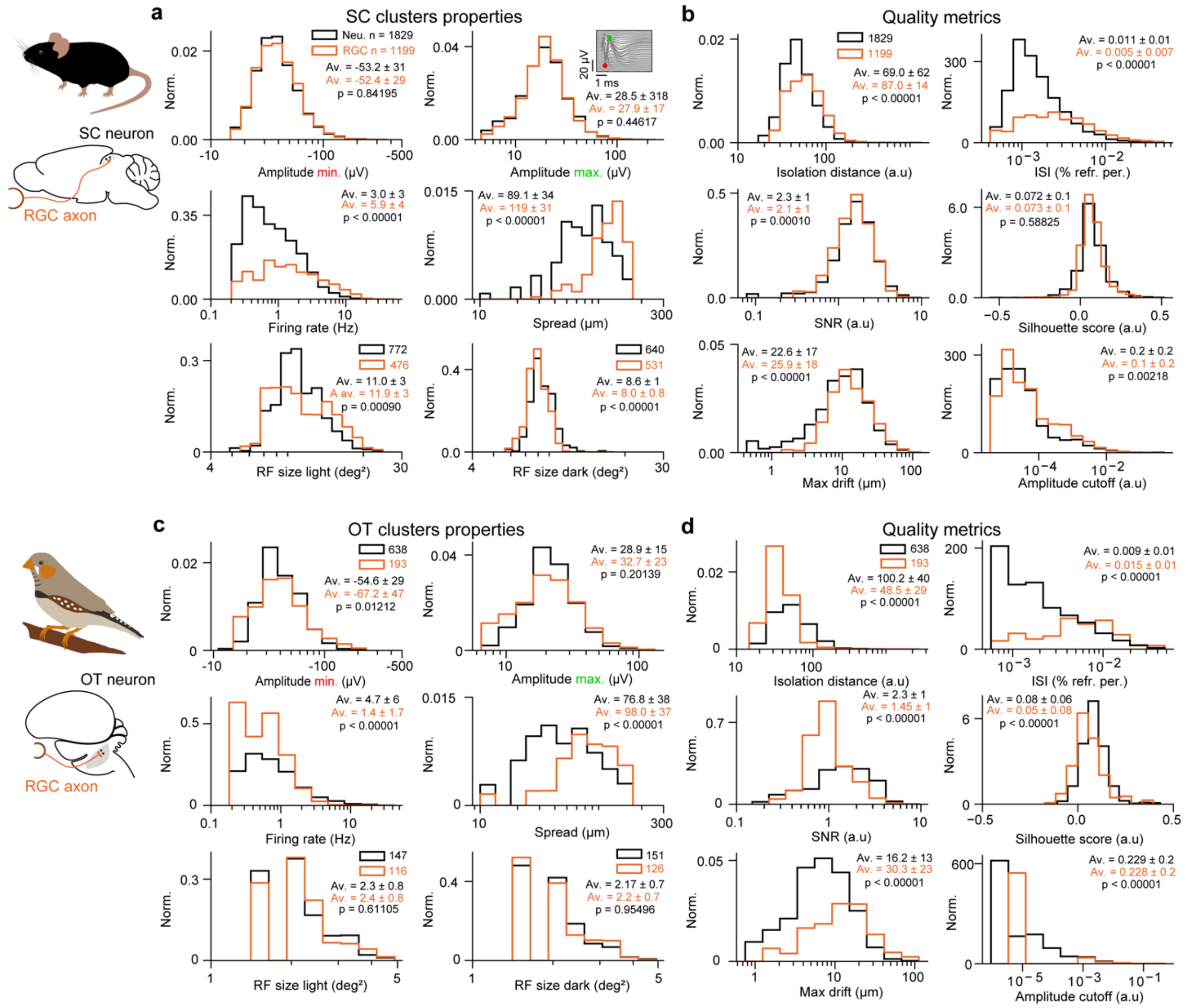

Fig. S4. Properties and quality metrics of single units in the mouse superior colliculus and zebra finch optic tectum. a-b Overview of the mouse dataset. **c-d** Overview of the zebra finch dataset. **a** Cluster properties in the mouse (n = 1840 SC neurons and n = 1199 RGC axons): Negative (top left) and positive (top right) amplitudes are estimated from the multi-channel-waveform, all shown on log-normal scale. Mean firing rate (FR) across the entire duration (middle left) and waveform spatial spread (middle right) in both populations of RGC axons and SC neurons show significant differences: RGC axons exhibit a higher FR and a larger waveform spread. Receptive field (RF) sizes, obtained from only clusters with high signal-to-noise RFs (SNR > 10) were estimated using both light (left, n = 476 RGCs and n = 772 SC neurons) and dark (right, n = 643 RGC axons and n = 531 SC neurons) sparse noise. p = 0.84/0.44/1.5×10^{-91}/6.9×10^{-114}/8.9×10^{-4} using two-sided Wilcoxon rank-sum test. **b** Single unit quality metrics. RGC axons and SC neurons have similar quality measures. p = 2.4×10^{-15}/5.3×10^{-33}/4.3×10^{-86}/1.4×10^{-16}/0.5/6.3×10^{-5}/0.0049), two-sided Wilcoxon rank-sum test. **c** Cluster properties in the zebra finch dataset (n = 638 OT neurons and n = 193 RGC axons), same format as in a. p = 0.01/0.20/3.7×10^{-17}/1.17×10^{-11}. Light sparse noise: n = 147 OT neurons and 116 RGC axons, p = 0.61. Dark sparse noise: n = 151 OT neurons and n = 126 RGC axons, p = 0.61. Two-sided Wilcoxon rank-sum test. **d** Quality metrics in the finch dataset, shown as in b. p = 0.95/3.8×10^{-21}/6.9×10^{-17}/1.6×10^{-16}/5.6×10^{-10}/3.3×10^{-23}/1.2×10^{-8}. Two-sided Wilcoxon rank-sum test. Quality metrics estimated using the ecephys modules (https://github.com/AllenInstitute/ecephys_spike_sorting). Source data are provided as a Source Data file.

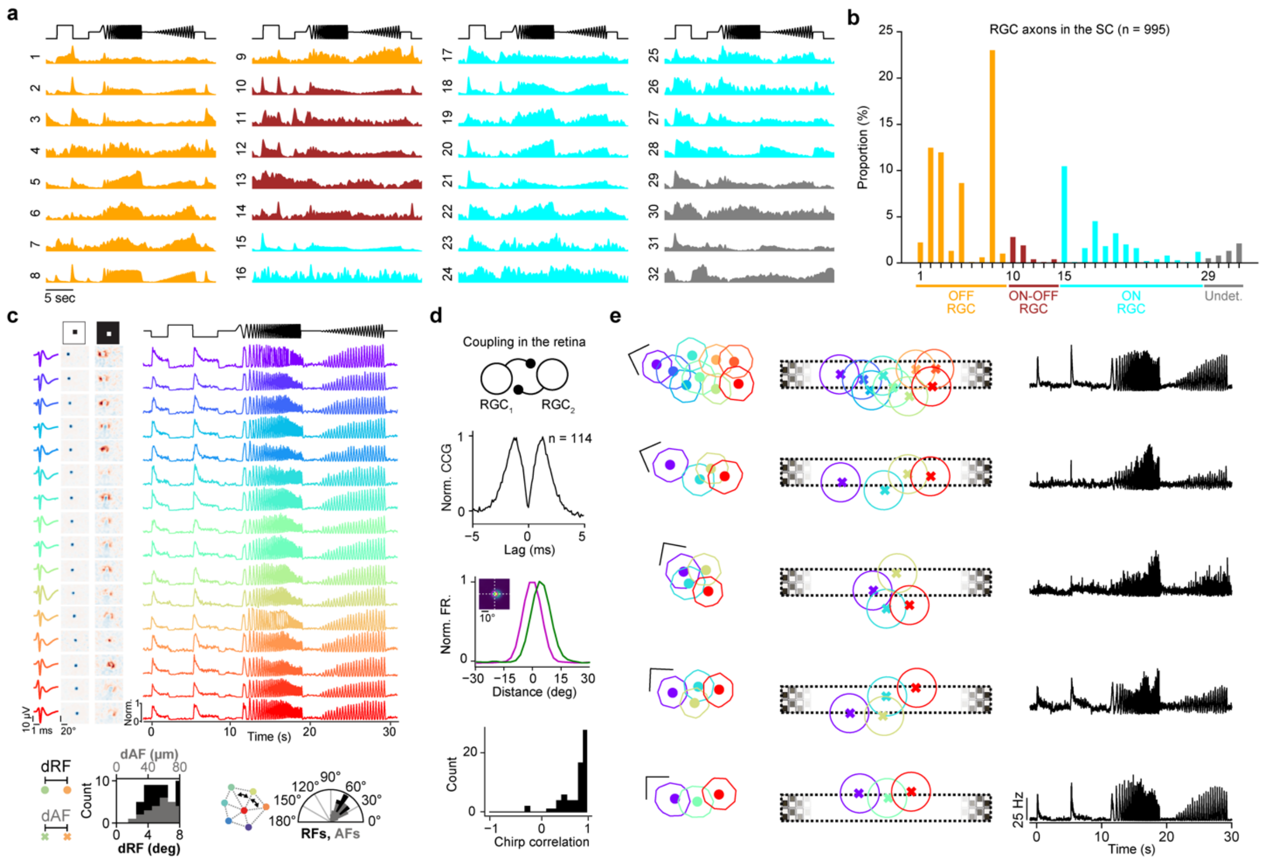

Fig. S5. Functional diversity and spatial organization of RGC axons within SC. a Diversity of recorded RGC axons within mouse SC. The RGC axons are sorted into one of the 32 RGC classes reported in[2] employing a correlation analysis adapted from[5]. The average responses in each class are shown. Note that RGC axons in the dataset cover a large diversity of RGC types. **b** Proportion of RGC axons measured in this study associated with the RGC classes from[2]. **c** Simultaneously recorded RGC axons with similar functional responses (OFF-transient). The receptive fields and axonal fields of this example are shown in Fig. 2c (left). This figure illustrates the waveforms at the best channels (left column), Receptive fields for both dark (middle left column) and light (middle right column) sparse noise targets, and the responses to the chirp stimulus (right). Note the classical OFF-center, ON-surround RF organization and the OFF-transient responses during the chirp stimulus. The receptive field centers and axonal field centers in this example exhibit properties that are characteristic for retinal mosaics: a gap at close distance (bottom left) and nearest neighbor angles between neighboring RGCs of around 60 deg (bottom right). The nearest neighbor angles between RFs and AFs were estimated by Delaunay triangulation and Voronoi tessellations[6]. **d** Double-peaked cross-correlograms identify putatively coupled RGCs. The average cross-correlogram of coupled RGCs from multiple experiments is shown (top, n = 114 RGC pairs, n = 32 mice). Putatively coupled RGC pairs are neighbors in the retina, evident by the close proximity of their receptive field centers (middle, RF distance = 4.70±1.95 °, n = 114 RGC pairs). The average receptive fields profiles of coupled RGC pairs are shown (green and magenta lines). The majority of coupled RGCs show similar functional responses to a chirp stimulus (bottom, correlation = 0.79±0.22, n = 114 coupled pairs). **e** Multiple examples of RGC receptive fields (left) and RGC axonal fields (middle) together with the average chirp responses (right) measured in mouse SC (n = 4 mice). The examples are also shown in Fig. 2 and shown here again to illustrate the functional responses of the RGCs. Scale bars for the RFs: 10 deg. Source data are provided as a Source Data file.

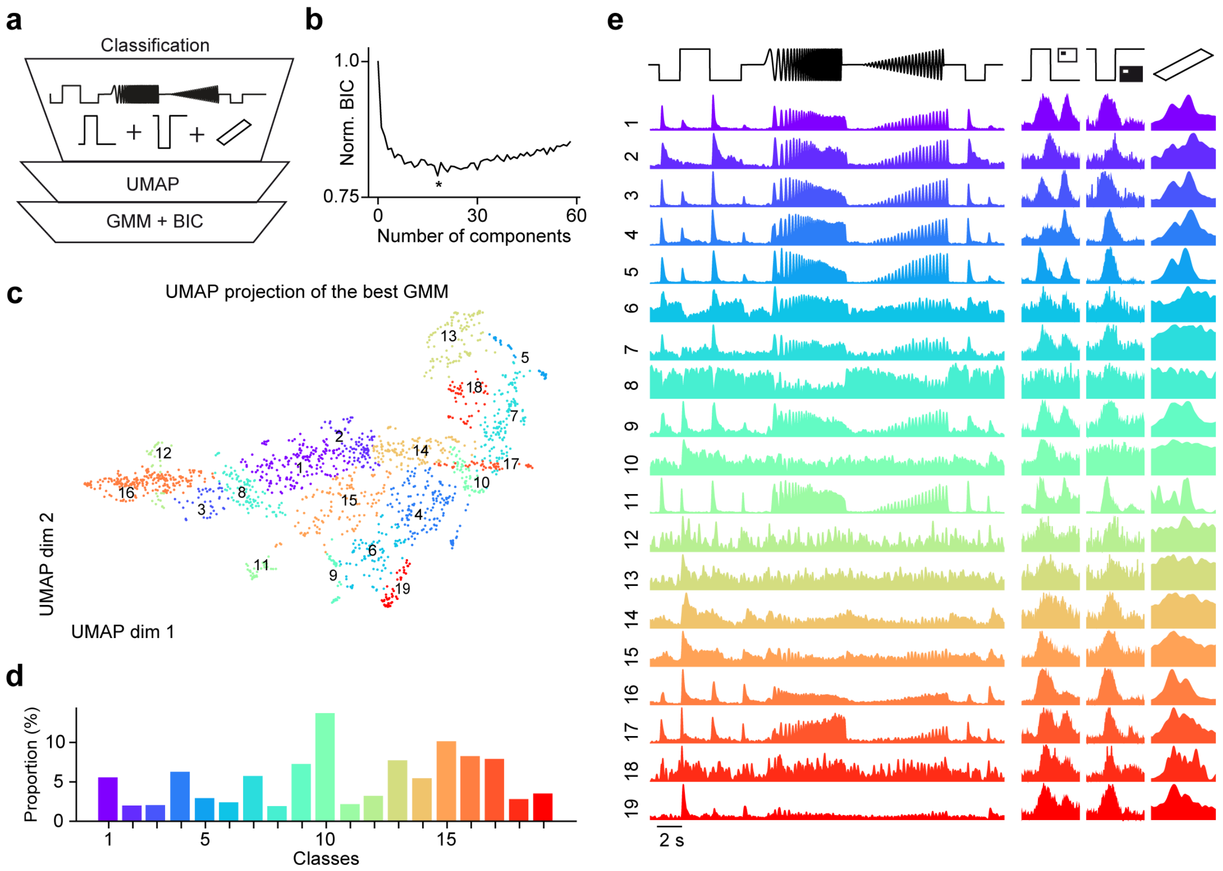

Fig. S6. Functional diversity of SC neurons. a Classification approach: 1) the evoked responses to the chirp stimulus, the sparse noises (black and white) and the moving bars were concatenated for individual SC neurons. 2) UMAP was used to reduce the dimensionality. 3) A Gaussian mixture model (GMM) was applied to the two-dimensional UMAP projection to identify functional SC groups. The GMM was performed multiple times with cluster numbers ranging from 1 to 60. **b** Bayesian Information Criterion (BIC) for each cluster number. Note, the cluster number minimizing the BIC was selected as the optimal cluster number (n = 19 clusters). **c** Two-dimensional UMAP projection of the GMM-classified dataset in class-color-code with n = 19 clusters. **d** Histogram showing the proportion of SC neurons in each class. **e** Identified functional SC neuron groups. The stimuli used to characterize the functional responses are shown on the top. The averaged evoked responses for each group are represented by the colors used in c and d. Source data are provided as a Source Data file.

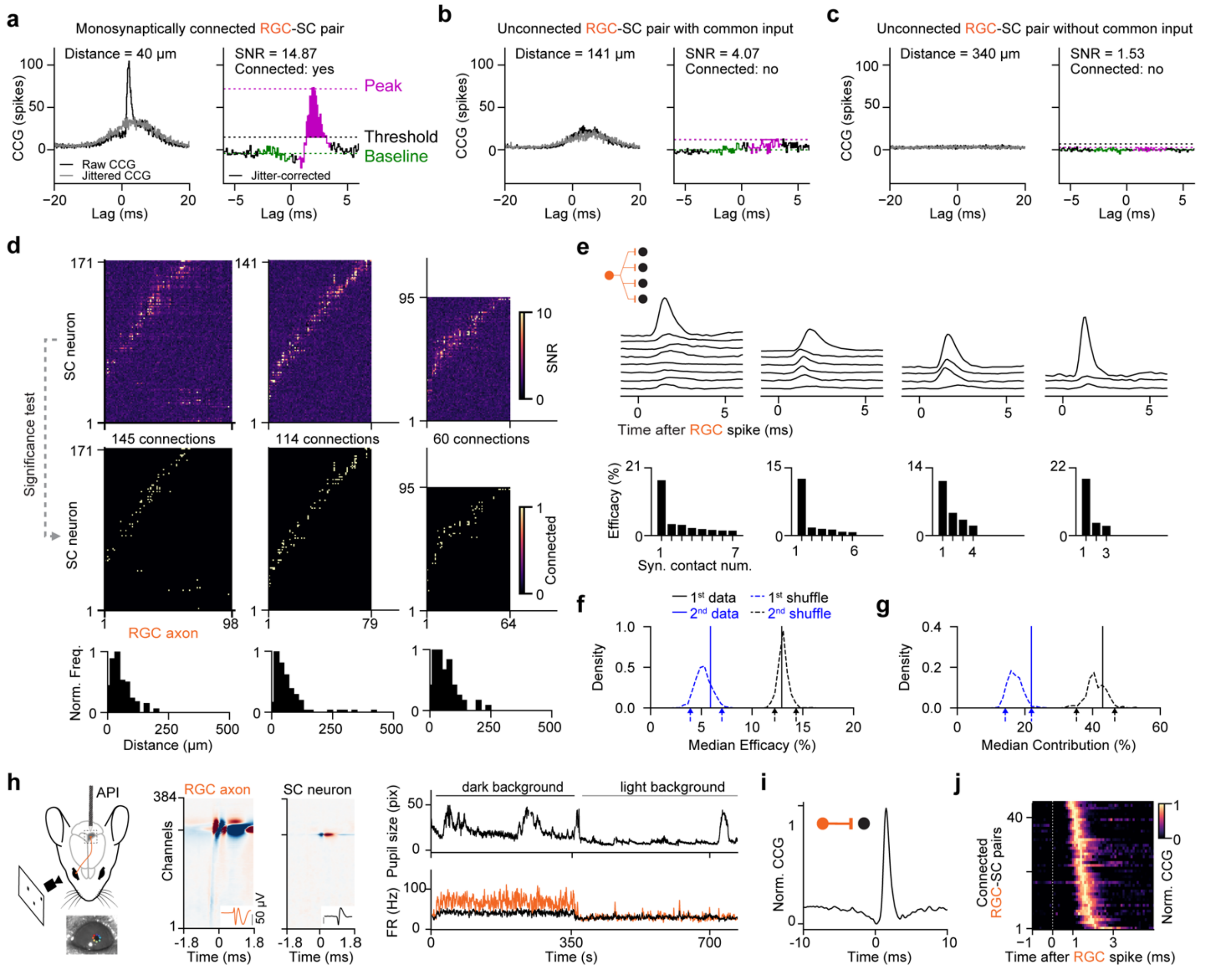

Fig. S7. In vivo connectivity analysis. a Example cross-correlogram (CCG) of a monosynaptically connected RGC-SC pair. Left, the black trace shows the raw CCG and the gray trace the jittered CCG. The transient peak at short latency is characteristic of monosynaptically connected neurons while the broader peak is common input. In the jitter-corrected CCG (raw-jitter) common input is subtracted to identify monosynaptically connected pairs (right) by estimating the statistical significance of the transient peak (magenta, see Methods). **b-c** Examples of unconnected RGC-SC pairs, with common input (b) and without common input (c). **d** Connectivity matrices of multiple recordings: the CCG peak SNR (top) and statistically identified connections (middle) are shown. The histograms of the distances between connected RGC-SC pairs on the probe are shown on the bottom. **e** Example CCGs of divergent connections from single RGC axons to multiple SC neurons (top). The efficacy is non-uniform with few strong and several weak connections (bottom). **f** Permutation test of the non-uniform connection strengths of divergent connections. Shown is the median efficacy of the data (solid line) and the distribution of the median efficacies of shuffled data (dashed line, n = 1000 repeats). The arrows indicate the 2.5% and 97.5% percentile intervals of the shuffled data distributions. **g** Same format as f but for the connection contribution. Note that the median of the 2nd is close to the 97.5% percentile point of the shuffled data (median data = 21.91 %, 97.5% point of shuffled data = 21.94 %). **h** Recording RGC axons in SC of awake mice. The recording configuration used to record from awake SC together with an example image of the pupil tracking is shown. The circles on the eye images schematically indicate the markers used for pupil position and size estimation. Example RGC axon and SC neuron recorded in awake mouse SC (middle). Pupil size during different stimulus conditions (right top) and the neuronal activity (right bottom) of an RGC axon (orange) and an SC neuron (black). **i** Example of a monosynaptically connected RGC-SC pair in an awake mouse. **j** CCGs of connected pairs across multiple experiments in awake mice (n = 45 connected pairs from n = 3 mice), sorted by peak latency. Source data are provided as a Source Data file.

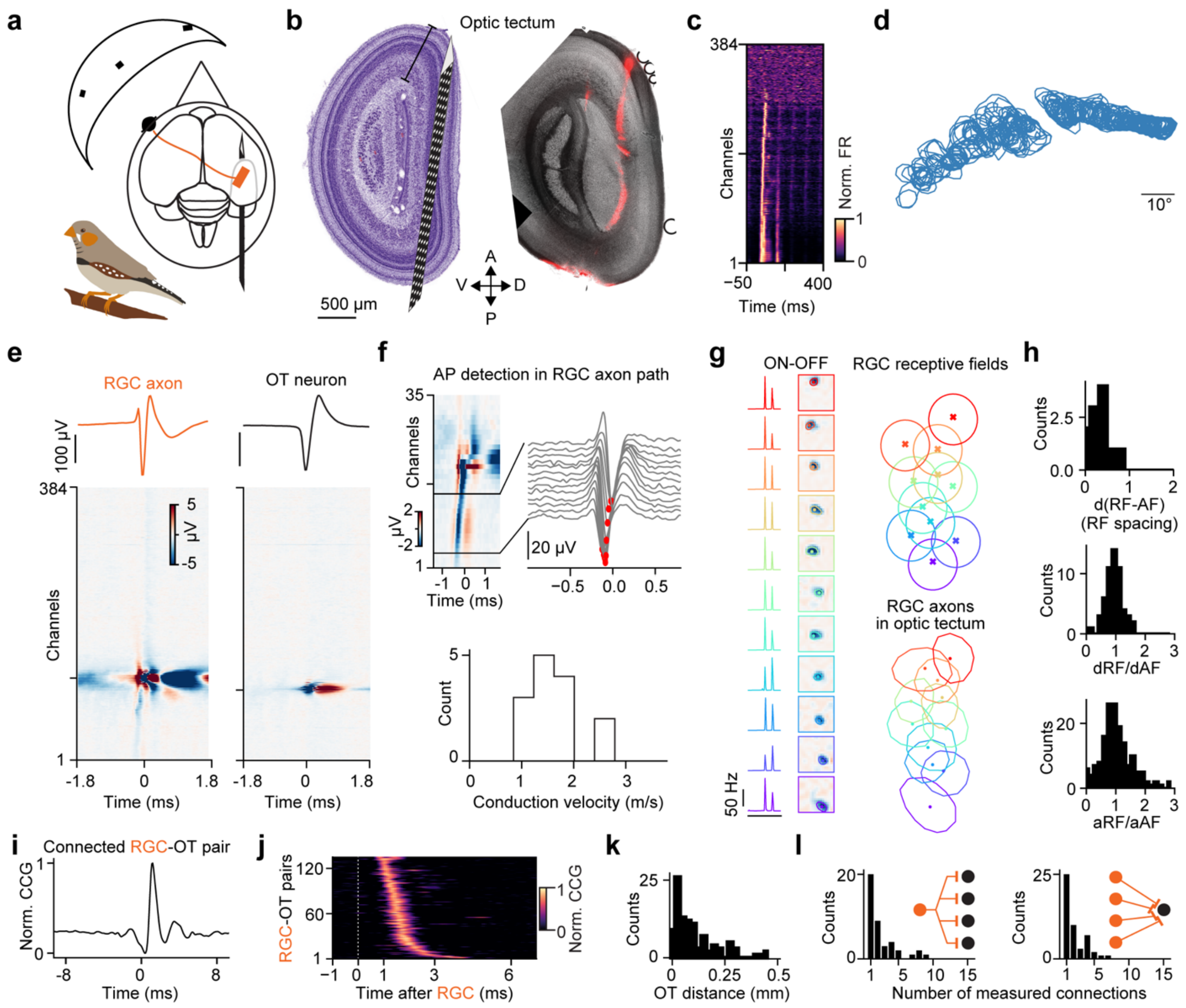

Fig. S8. Studying retinotectal connections in zebra finches. a Schematic of the retinotectal projections in the zebra finch and of the recording configuration. **b** Sagittal slices of the optic tectum (OT) with DiI staining from an antero-posterior insertion (right), with its approximate corresponding location in the ZEBrA histological atlas (left). **c** Visually-evoked multi-unit-activity identifies the recording sites located within the optic tectum. The colormap shows the PSTH of each channel in response to a sparse noise stimulus. **d** Receptive fields of the multi-unit-activity shown in c. Please note the gap in the receptive fields which is due to a sudden jump in retinotopy across multiple recording sites. **e** Example of an RGC axon and an OT neuron recorded with a high-density electrode in zebra finch *in vivo*. **f** Top, closeup view on an RGC axon measured in the OT. The traces show the detection of the action potential in the axon path by the local minimum (red dots). Bottom, axonal conduction velocities across multiple recordings and RGC axons (mean conduction velocity = 1.5±0.5 m/s, n = 14 RGC axons, n = 2 zebra finches). **g** Simultaneously recorded RGC axons with similar functional response properties, characterized using the chirp stimulus and receptive fields. In this example RGCs were from the ON-OFF type. The corresponding receptive fields (RF, top right) and axonal fields (AF, bottom right) are similar in their spatial organization and form. **h** Top, histogram of the distances between RF and AF centers shown in g in the unit of RF spacing (median RF-AF distance = 0.41±0.21 RF spacings, n = 11 RGCs). Middle, distances between RF centers (dRF) and AF centers (dAF) are similar. Histogram of the dRF/dAF ratios (median dRF/dAF = 1.01±0.28, n = 55 RGC pairs). Bottom, angles between RGC triples are similar for RFs and AFs. Histogram of the αRF/αAF ratios (median αRF/αAF = 1.03±0.76, n = 166 angles). **i** Example of an identified connection between an RGC axon RGC and an OT neuron. Shown is the spike train cross-correlogram. **j** Colormap showing the cross-correlation of all connected RGC-OT pairs recorded in the zebra finch dataset (n = 3 zebra finches). **k** Distances between connected RGC-OT pairs. n = 105 connected RGC-OT pairs. **l** Histogram showing the number of divergent and convergent RGC-OT connections. Source data are provided as a Source Data file.

Supplementary References

1. Sibille, J., Gehr, C., Teh, K. L. & Kremkow, J. Tangential high-density electrode insertions allow to simultaneously measure neuronal activity across an extended region of the visual field in mouse superior colliculus. *J Neurosci Meth* 109622 (2022)

2. Baden, T. *et al.* The functional diversity of retinal ganglion cells in the mouse. *Nature* 529, 345–350 (2016).

3. Steinmetz, N. A., Zatka-Haas, P., Carandini, M. & Harris, K. D. Distributed coding of choice, action and engagement across the mouse brain. *Nature* 576, 266–273 (2019).

4. Shamash, P., Carandini, M., Harris, K. D. & Steinmetz, N. A. A tool for analyzing electrode tracks from slice histology. *Biorxiv* 447995 (2018)

5. Rosón, M. R. *et al.* Mouse dLGN Receives Functional Input from a Diverse Population of Retinal Ganglion Cells with Limited Convergence. *Neuron* 102, 462-476.e8 (2019).

6. Zhan, X. J. & Troy, J. B. Modeling cat retinal beta-cell arrays. *Visual Neurosci* 17, 23–39 (2000).
