# Supplementary material for: High-density electrode recordings reveal strong and specific connections between retinal ganglion cells and midbrain neurons
Source: Nat Commun. 2022 Sep 5;13:5218. doi: 10.1038/s41467-022-32775-2 (PMC9445019; doi:10.1038/s41467-022-32775-2)
Supplement: Supplementary file 3 — Reporting Summary [file 41467_2022_32775_MOESM3_ESM.pdf]

nature portfolio

Corresponding author(s): Jens Kremkow

Last updated by author(s): 08/02/822

Reporting Summary

Nature Portfolio wishes to improve the reproducibility of the work that we publish. This form provides structure for consistency and transparency in reporting. For further information on Nature Portfolio policies, see our Editorial Policies and the Editorial Policy Checklist.

Statistics

For all statistical analyses, confirm that the following items are present in the figure legend, table legend, main text, or Methods section.

n/a	Confirmed	
☐	☒	The exact sample size (*n*) for each experimental group/condition, given as a discrete number and unit of measurement
☐	☒	A statement on whether measurements were taken from distinct samples or whether the same sample was measured repeatedly
☐	☒	The statistical test(s) used AND whether they are one- or two-sided<br>*Only common tests should be described solely by name; describe more complex techniques in the Methods section.*
☐	☒	A description of all covariates tested
☒	☐	A description of any assumptions or corrections, such as tests of normality and adjustment for multiple comparisons
☐	☒	A full description of the statistical parameters including central tendency (e.g. means) or other basic estimates (e.g. regression coefficient) AND variation (e.g. standard deviation) or associated estimates of uncertainty (e.g. confidence intervals)
☐	☒	For null hypothesis testing, the test statistic (e.g. *F*, *t*, *r*) with confidence intervals, effect sizes, degrees of freedom and *P* value noted<br>*Give P values as exact values whenever suitable.*
☒	☐	For Bayesian analysis, information on the choice of priors and Markov chain Monte Carlo settings
☒	☐	For hierarchical and complex designs, identification of the appropriate level for tests and full reporting of outcomes
☐	☒	Estimates of effect sizes (e.g. Cohen's *d*, Pearson's *r*), indicating how they were calculated

Our web collection on statistics for biologists contains articles on many of the points above.

Software and code

Policy information about availability of computer code

Data collection	Kilosort 2 & 2.5/ Open Ephys v0.5.5.2/Phy 2/Python 2 & 3/Matlab 2018 & 20219
Data analysis	Python 2 & 3/Matlab 2018 & 2019/DeepLabCut 2.1

For manuscripts utilizing custom algorithms or software that are central to the research but not yet described in published literature, software must be made available to editors and reviewers. We strongly encourage code deposition in a community repository (e.g. GitHub). See the Nature Portfolio guidelines for submitting code & software for further information.

Data

Policy information about availability of data

All manuscripts must include a data availability statement. This statement should provide the following information, where applicable:

- Accession codes, unique identifiers, or web links for publicly available datasets
- A description of any restrictions on data availability
- For clinical datasets or third party data, please ensure that the statement adheres to our policy

The data shown in the figures are provided in the Source Data file. A minimum dataset for illustrating RGC axons in Neuropixels recordings in mouse SC has been deposited in a repository on Zenodo. The raw Neuropixels datasets generated in this study are too large to made available online and are available upon request from the correspondence author (jens.kremkow@charite.de).

Human research participants

Policy information about studies involving human research participants and Sex and Gender in Research.

Reporting on sex and gender	N/A
Population characteristics	N/A
Recruitment	N/A
Ethics oversight	N/A

Note that full information on the approval of the study protocol must also be provided in the manuscript.

Field-specific reporting

Please select the one below that is the best fit for your research. If you are not sure, read the appropriate sections before making your selection.

☒ Life sciences ☐ Behavioural & social sciences ☐ Ecological, evolutionary & environmental sciences

For a reference copy of the document with all sections, see nature.com/documents/nr-reporting-summary-flat.pdf

Life sciences study design

All studies must disclose on these points even when the disclosure is negative.

Sample size	The sample size was limited to reduce the number of used animals and to keep enough statistical power as predefined in ethical approvals. The minimal effect size was predetermined upon acknowledgments with the authority and confirmed based the obtained confidence intervals.
Data exclusions	Data were included based on signal-to-noise and goodness-of-fit criteria to obtain accurate measurements. For each analysis, responsiveness to the visual stimulus was estimated as indicated and used to excluded non-responsive neurons or axons with the given signal-to-noise thresholds. Axonal fields were included when the fit with a two-dimensional Gaussian function had an R2 > 0.8. Single unit clusters produced by Kilosort were recheck individually in the pharmacological experiments to exclude waveforms exhibiting different form from one pharmacological condition to the next.
Replication	The main findings were replicated in individual experiments, e.g. axonal waveforms were identified in individual well targeted recordings in both mice and zebra finches. Connections between retinal ganglion cells and SC/OT neurons could be identified in experiments that contained axonal waveforms. The precise spatial organization of retinal ganglion cell receptive fields and axonal fields could be reproduced in at least n = 5 mice and n = 2 zebra finches.
Randomization	The presentation of the visual stimuli was randomized. Mice were not randomized as they all stem from controlled C57BL6/J background, obtained from the Charité animal facility. Likewise, zebra finches were not randomized. The analysis shown in Figure S7f/g involved a permutation test.
Blinding	All recordings performed in the superior colliculus of mice and optic tectum of finches require a detailed (non-blinded) examination of all probed parameters.

Reporting for specific materials, systems and methods

We require information from authors about some types of materials, experimental systems and methods used in many studies. Here, indicate whether each material, system or method listed is relevant to your study. If you are not sure if a list item applies to your research, read the appropriate section before selecting a response.

Materials & experimental systems

n/a	Involved in the study	
☒	☐	Antibodies
☒	☐	Eukaryotic cell lines
☒	☐	Palaeontology and archaeology
☐	☒	Animals and other organisms
☒	☐	Clinical data
☒	☐	Dual use research of concern

Methods

n/a	Involved in the study	
☒	☐	ChIP-seq
☒	☐	Flow cytometry
☒	☐	MRI-based neuroimaging

Animals and other research organisms

Policy information about studies involving animals; ARRIVE guidelines recommended for reporting animal research, and Sex and Gender in Research

Laboratory animals	Mice from 8 to 24 weeks (C57BL6/J) were bred at 24°C between 60-70% humidity at the local breeding facility (Charité-Forschungseinrichtung für Experimentelle Medizin) or ordered from Charles-River Germany. They were housed in 12 hours dark/light cycles and provided with food and water ad-libitum. Adult male zebra finches (>180 days post-hatch) were bred at the Max Planck Institute for Ornithology in Seewiesen, housed in 14/10-hour light/dark cycles at 24°C between 60-70% humidity, and provided with food and water ad-libitum.
Wild animals	No wild animals were used in this study
Reporting on sex	All animals used were male mice or male zebra finches as no clear indication suggests that visual transmission and processing between the retinal and the Superior Colliculus (or the Optic Tectum) is subject to sexual dimorphism; until further examinations would indicate otherwise.
Field-collected samples	There were no field samples collected in this study.
Ethics oversight	All experiments were pursued in agreement with the local authorities upon defined procedures.<br>Mouse: Landesamt für Gesundheit und Soziales Berlin - G0142/18<br>Finches: Regierungspräsidium Oberbayern - ROB-55. 2-2532. VET_02-18-182

Note that full information on the approval of the study protocol must also be provided in the manuscript.
